# Suppression of Spry4 enhances cancer stem cell properties of human MDA-MB-231 breast carcinoma cells

**DOI:** 10.1186/s12935-016-0292-7

**Published:** 2016-03-11

**Authors:** Hongyu Jing, Lucy Liaw, Robert Friesel, Calvin Vary, Shucheng Hua, Xuehui Yang

**Affiliations:** Center for Molecular Medicine, Maine Medical Center Research Institute, 81 Research Drive, Scarborough, ME 04074 USA; Department of Respiratory Medicine, The First Hospital of Jilin University, Changchun, 130021 Jilin Province China

**Keywords:** Sprouty4 (Spry4), Cancer stem cells, Beta3- integrin (CD61), CD133, Receptor tyrosine kinases (RTK)

## Abstract

**Background:**

Cancer stem cells contribute to tumor initiation, heterogeneity, and recurrence, and are critical targets in cancer therapy. Sprouty4 (Spry4) is a potent inhibitor of signal transduction pathways elicited by receptor tyrosine kinases, and has roles in regulating cell proliferation, migration and differentiation. Spry4 has been implicated as a tumor suppressor and in modulating embryonic stem cells.

**Objectives:**

The purpose of this research was to test the novel idea that Spry4 regulates cancer stem cell properties in breast cancer.

**Methods:**

Loss-of function of Spry4 in human MDA-MB-231 cell was used to test our hypothesis. Spry4 knockdown or control cell lines were generated using lentiviral delivery of human Spry4 or non-targeting control shRNAs, and then selected with 2 μg/ml puromycin. Cell growth and migratory abilities were determined using growth curve and cell cycle flow cytometry analyses and scratch assays, respectively. Xenograft tumor model was used to determine the tumorigenic activity and metastasis in vivo. Cancer stem cell related markers were evaluated using immunoblotting assays and fluorescence-activated cell sorting. Cancer stem cell phenotype was evaluated using in vitro mammosphere formation and drug sensitivity tests, and in vivo limiting dilution tumor formation assay.

**Results:**

Two out of three tested human Spry4 shRNAs significantly suppressed the expression of endogenous Spry4 in MDA-MB-231 cells. Suppressing Spry4 expression increased MDA-MB-231 cell proliferation and migration. Suppressing Spry4 increased β3-integrin expression, and CD133^+^CD44^+^ subpopulation. Suppressing Spry4 increased mammosphere formation, while decreasing the sensitivity of MDA-MB-231 cells to Paclitaxel treatment. Finally, suppressing Spry4 increased the potency of MDA-MB-231 cell tumor initiation, a feature attributed to cancer stem cells.

**Conclusions:**

Our findings provide novel evidence that endogenous Spry4 may have tumor suppressive activity in breast cancer by suppressing cancer stem cell properties in addition to negative effects on tumor cell proliferation and migration.

**Electronic supplementary material:**

The online version of this article (doi:10.1186/s12935-016-0292-7) contains supplementary material, which is available to authorized users.

## Background

Breast cancer is the most common cancer among women, and despite tremendous advances in diagnosis and treatment at an early stage, it is still the second leading cause of cancer related deaths among women in the United States [1]. Recurrence and metastasis of the primary tumor are thought to be key contributors to the incurable nature of metastatic breast cancer. Accumulating evidence suggests that tumor recurrence, metastasis and poor clinical outcome of cancer patients is strongly influenced by a small subset of stem-like cells, also called cancer stem cells (CSCs) [2–4]. CSCs are tumor initiating cells that evade the effects of systemic therapies. They have the capacity to self-renew and differentiate into bulk tumor cells, and demonstrate resistance to standard chemotherapy [3, 4]. Despite the recognition that CSCs are a critical target for tumor eradication, the molecular regulators of CSC phenotype remain poorly understood.

Receptor tyrosine kinases (RTK) play central roles in multiple biological processes including proliferation, survival, differentiation and migration [5], and are often associated with normal and CSC identity mainly through activating Ras/ERK and PI3K/Akt signaling pathways [6–8]. Spry4 is a feedback regulator that is induced by RTK/MAPK kinase and restrains RTK signaling output. Spry4 displays tumor suppressor activity by inhibiting tumor cell migration and proliferation in human cancers including lung [9], prostate [10] and breast cancers [11]. This study tests the novel idea that Spry4 regulates properties of the CSC in breast carcinoma. We used lentiviral delivery of human Spry4 shRNAs to suppress endogenous Spry4 expression in human MDA-MB-231 cells, and found this efficiently altered the phenotype of the CSC subpopulation, leading to a more malignant and drug-resistant phenotype. Our studies suggest that the endogenous activity of Spry4 targets CSCs to promote the tumor suppressive phenotype.

## Methods

### Cell culture

MDA-MB-231 breast cancer cell from ATCC were cultured in α-MEM containing 10 % FBS supplemented with 1 % non-essential amino acids (invitrogen) and penicillin/streptomycin/amphotericin B. To generate stable Spry4 knockdown cells, low passage MDA-MB-231 cells (passage 10–15) were transduced with human Spry4 shRNA lentiviruses or non-targeting control lentiviruses (Open Biosystems), and selected in medium containing 2 μg/ml puromycin.

### Western blotting

Cells were lysed in HNTG buffer [20 mM HEPES pH7.4, 150 mM NaCl, 10 % glycerol, 1 % Triton X-100, 1.5 mM MgCl2, 1.0 mM EGTA and proteinase inhibitor cocktail (Roche)]. Cell lysates were subjected to SDS-PAGE separation. Immunoblotting was performed with antibodies against Spry4, EGFR, ERK, β1-integrin, β3-integrin and Src (Santa Cruz), phosphor-ERK, phosphor-Akt, Akt, pSrc (Cell Signaling), and tubulin (Sigma).

### Cell growth curve analysis and anchorage-independent colony forming assay

Spry4 knockdown (S4kd) or non-targeting (NT) control stable cell lines were trypsinized and counted. For growth curve analysis, 5 × 10^3^ S4kd or NT stable cells were plated in each well of 12 well plates in triplicate, cultured in growth media, and counted by Coulter counter (Beckman Coulter, Inc.). For anchorage-independent colony formation, 1 × 10^5^ S4kd or NT stable cells were mixed with medium containing 0.4 % agar and were spread on top of a bottom agar layer (0.8 % agar in growth medium). Cells were grown for 2 weeks, and colonies were counted and photographed. The diameter of the colonies was measured using Image J software (NIH).

### Mammosphere assay

Mammosphere assays were performed as described by Dontu [12] with modifications. Briefly, 5000 of NT or S4kd cells were suspended in serum-free αMEM containing 20 ng/ml FGF2, 20 ng/ml EGF (R&D Systems) and 1xB27 serum free supplement (invitrogen) and cultured on ultra-low attachment 6-well plates. Mammospheres were monitored daily by phase-contrast microscopy to ensure that they were derived from a single cell. The number of mammospheres was counted at day 10, and their size was measured using ImageJ (NIH).

### Fluorescence-activated cell sorting (FACS) analysis

NT and S4kd cells were trypsinized, stained with fluor- conjugated antibodies: anti-CD61-APC, anti-CD29-PE, anti-CD133-PE, anti-CD24-FITC, anti-CD49f-FITC and anti-CD44-APC, and analyzed on FACSCalibur (BD Biosciences). The data were analyzed using FlowJo software.

### Scratch assay

NT and S4kd cells were plated in 6 well plates in triplicate at subconfluence and cultured for 24 h. Confluent cells were treated with 2 μg/ml mitomycin C for 2 h prior to cell denudation using a 1 ml pipette tips. Cells were washed with growth medium and continually cultured in growth medium containing 1 μg/ml mitomycin C for 48 h. The progress of migration was photographed in eight regions at 0, 24 and 48 h. Denuded areas were measured and quantified with Image J.

### Animal xenograft analysis

Six to eight-week old NOD/SCID female mice (Jackson Laboratory) were used for xenograft tumor studies according to previous report. NT or S4kd MDA-MB-231 stable cells were harvested in the exponential growth phase using EDTA solution and washed twice with ice cold PBS, and resuspended in PBS at the dose of 1 × 10^6^ per 200 ul. 200 ul of cells were injected into the left inguinal mammary fat pad, five mice were used per cell line. Tumor length and width was measured with a caliper weekly, and tumor volume calculated using the formula W^2^L/2 (L = length, W = width) [13]. Nine weeks later when tumors were approximately 10–15 mm at their largest diameter, tumors and lungs were removed and snap frozen or fixed in 10 % formalin for further analysis. For in vivo limiting dilution assay (LDA), mice were injected with 1 × 10^3^, 1 × 10^4^ or 1 × 10^5^ cells, and monitored daily. Tumor formation was verified at end-stage after 4 month after tumor cell injection. All procedures involving animals were approved by the Institutional Animal Care and Use Committee of Maine Medical Center, and conducted in compliance with regulatory guidelines involving the use of vertebrate animals in biomedical research.

### Statistical analysis

The results were presented as mean ± SD and analyzed with Student’s *t* test. *P* < 0.05 was denoted as statistically significant.

## Results

### Suppression of Spry4 in MDA-MB-231 cells promotes cell proliferation and migration in vitro

**Fig. 1:**
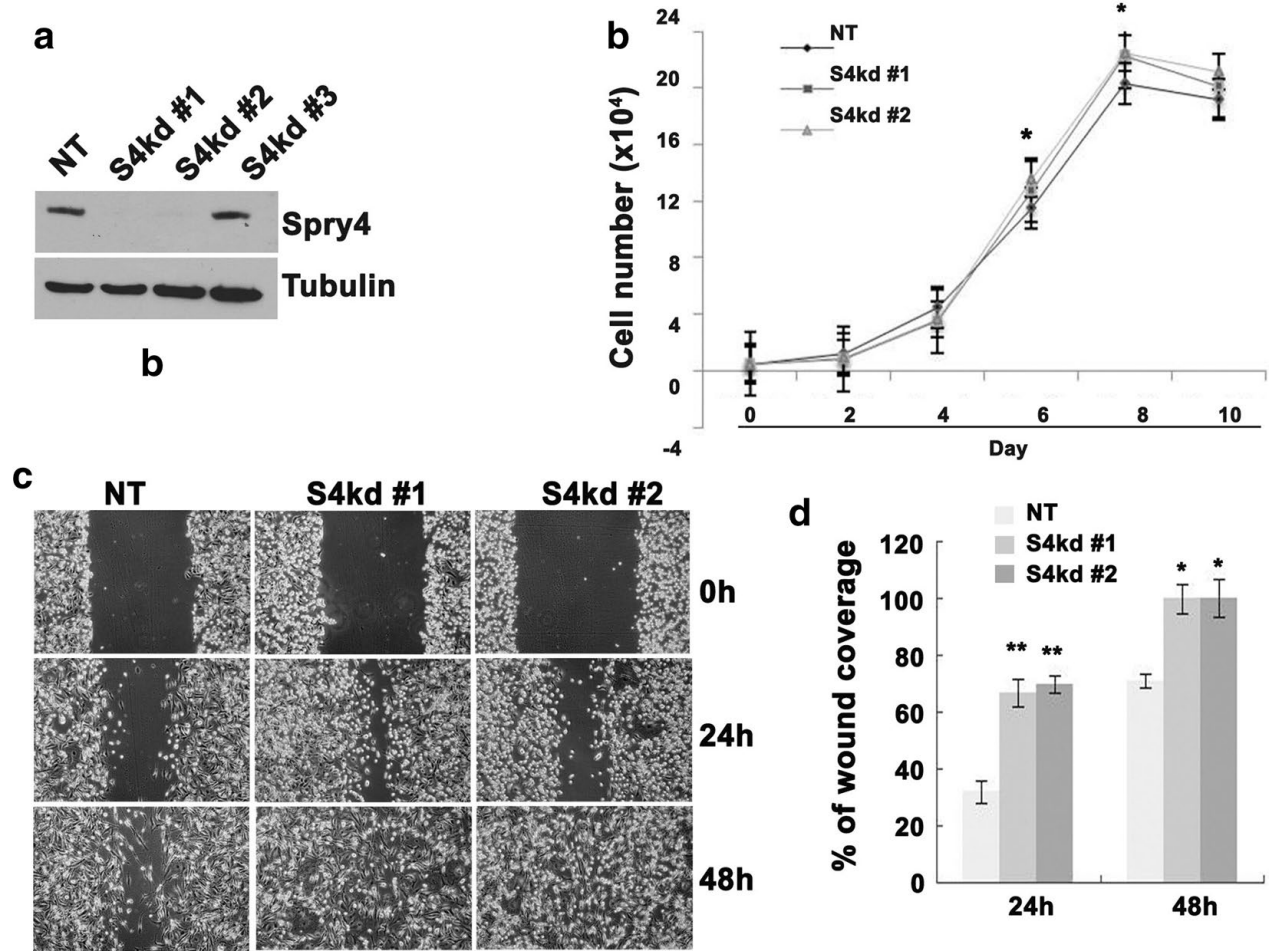
Suppressing Spry4 expression enhances MDA-MB-231 cell growth and migration. **a** Immunoblotting assay shows that two out of three Spry4 shRNAs effectively decreased Spry4 protein levels compared to NT control. **b** Growth curve analysis shows that suppressing Spry4 expression increased MDA-MB-231 cell growth. **c** Representative images of scratch assays from three independent experiments show that suppressing Spry4 expression increased cell migration into the denuded area. **d** Quantification of cell migration capacity from one of three experiments. *p < 0.05; **p < 0.01

MDA-MB-231 is a human breast cancer cell line that endogenously produces Spry4 protein (Fig. 1a). To examine the role of Spry4 in regulation of the malignant phenotype of these cells, we performed shRNA-mediated knockdown of human Spry4 compared to a non-targeting control. Stable knockdown of Spry4 (S4kd) and non-targeting control (NT) cell lines were obtained by puromycin selection. Three different shRNAs targeting Spry4 were utilized, and two of them efficiently reduced Spry4 protein to undetectable levels (S4kd#1 and S4kd#2) (Fig. 1a). Growth curve analyses showed that suppression of Spry4 led to an increase in cell number over a ten-day cell growth period (Fig. 1b). Cell cycle analyses confirmed that the increased growth by suppressing Spry4 associated with the increased cells in S and G2/M phases (Additional file 1). We also tested cell migration, since highly motile cells are associated with cancer metastasis. A scratch assay was used in the presence of mitomycin C to suppress cell proliferation. Cell migration into the denuded area was quantified at 24 and 48 h. Figure 1c, d show that knockdown of Spry4 increased cell migration, with closure of the denuded area more quickly than the control cells. These data show that loss of Spry4 increases both proliferation and migration in MDA-MB-231 cells, suggesting that endogenous Spry4 protein acts to suppress these activities.

### Suppression of Spry4 potentiates MDA-MB-231 cell in vitro anchorage-independent growth, and in vivo tumor growth and lung metastasis

**Fig. 2:**
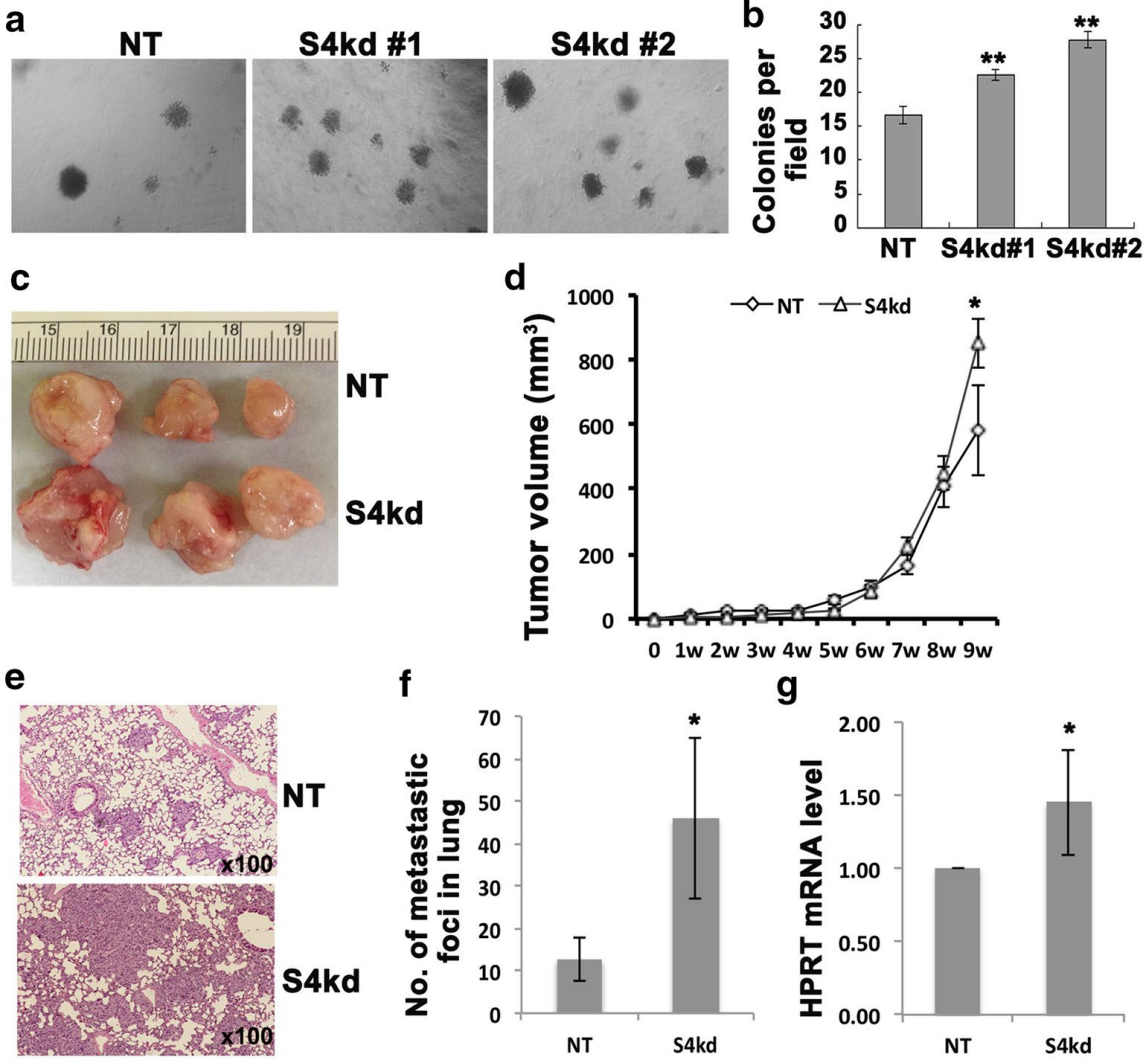
Suppressing Spry4 expression promotes MDA-MB-231 tumor growth and lung metastasis. **a** Representative images of soft-agar colony formation assays show that S4kd cells formed more colonies compared to NT cells. **b** Quantification of soft-agar colony formation assay. **c** Representative images of tumors harvested at 9 weeks after fat pad inoculation of 1 × 10^6^ NT or S4kd#1 cells. **d** Tumor growth curve was present with average tumor volume from five animals in each group. **e** Representative H&E staining of lungs from 1 × 10^6^ dosage xenograft mice showing more and larger metastasis lesions in S4kd injected mice compared to NT injected mice. **f** Quantification of lung metastasis. **g** RT-qPCR analysis of human HRPT transcript versus total 18S rRNA transcripts in lungs from S4kd and NT cells injected mice, the relative mRNA level of human HRPT in S4kd tumors compare to NT tumors is presented. *p < 0.05, **p < 0.01

Anchorage-independent growth is one of the fundamental features of malignant tumor cells. We examined the colony forming capacity of Spry4 knockdown cells in soft agar, and found that both Spry4 knockdown populations have increased colony number compared to non-targeting control, suggesting conversion into a more malignant phenotype (Fig. 2a, b).

To test whether the in vitro features of Spry4 knockdown cells are maintained in vivo, we performed orthotopic xenograft analysis to test if knockdown of Spry4 affects the tumor formation by injecting 1 × 10^6^ NT or S4kd#1 cells into the mammary fat pads of immunodeficient NOD/SCID mice. Tumor growth was monitored and measured weekly. All injected mice developed palpable tumors within 2 weeks. However, S4kd tumors grew to a greater final size compared to control tumors (Fig. 2c, d). Furthermore, mice with S4kd tumors had an increased rate of spontaneous lung metastases compared to mice bearing NT tumors. This was quantified by counting representative metastatic lung foci from H&E stained histological sections (Fig. 2e, f), and by using RT-qPCR to identify levels of human HPRT mRNA in the mouse lungs (Fig. 2g). Thus, the increased malignant phenotype due to loss of Spry4 was maintained in vivo in primary tumors as well as secondary, metastatic tumors.

### Suppression of Spry4 increases β3-intergin expression of MDA-MB-231 cells

**Fig. 3:**
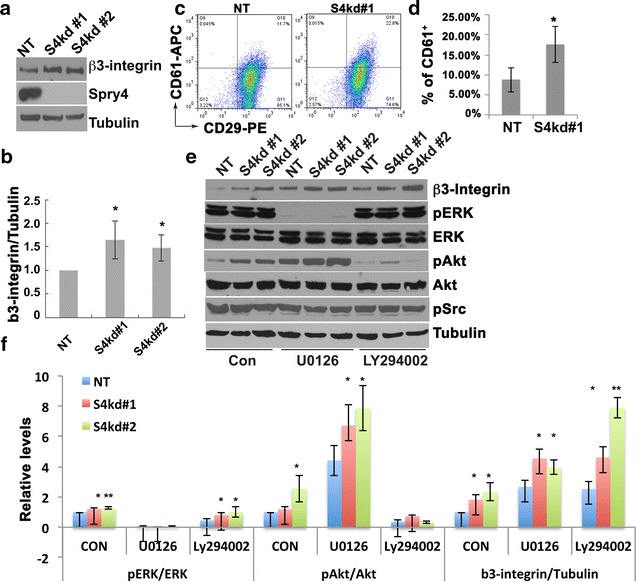
Suppressing Spry4 expression increases β3-integrin protein level in MDA-MB-231 cells. **a** Immunoblotting assay shows that S4kd cells had increased β3-integrin levels. **b** Quantification of β3-integrin protein levels from three independent experiments. **c** Representative FACS analysis shows that suppressing Spry4 expression increased CD61 positive cells. **d** Quantification of CD61 positive cell percentages from three independent experiments. **e** Immunoblotting assay shows that suppressing Spry4 had no effect on pERK and pSrc expression, but increased pAkt expression. Inhibition of MEK/ERK signaling by U0126 or PI3 K/Akt by Ly294002 did not reverse Spry4 knockdown mediated increase of β3-integrin but further increased β3-integrin expression. **f** Quantification of pERK, pAkt and b3-integrin from three independent immunoblotting assays in E. *p < 0.05; **p < 0.01

Integrins are the cell surface receptors that interact with ligands in the extracellular matrix (ECM), and play critical role in tumor growth and metastasis [14, 15]. We have previously shown that Spry4 regulates β3-integrin expression in endothelial cells [16]. Therefore, we tested whether suppressing Spry4 modulates β3-integrin expression in MDA-MB-231 cells. Immunoblotting analysis shows that knockdown of Spry4 increased β3-integrin protein level compared to control cells (Fig. 3a, b). FACS analyses using fluorescence-conjugated β1 (CD29) and β3-integrin (CD61) antibodies showed that suppressing Spry4 increased the CD61 positive cell population (Fig. 3c, d). It is noteworthy that almost all MDA-MB-231 cells are β1-integrin (CD29) positive cells (>85 %), and suppression of Spry4 had minimal effect on β1-integrin expression levels (Fig. 3c). Spry4 is a well-known inhibitor of RTK mediated MEK/ERK and PI3 K/Akt signaling pathways in multiple cell types [17–19], we examined the pERK, pAkt, as well as the pSrc expression in S4kd MDA-MB-231 cells cultured in growth medium. Suppressing Spry4 had significant increase of pAkt, mild but significant increase of pERK, but no effect on pSrc level. However, neither inhibition of MEK/ERK nor PI3K/Akt signaling reversed this up-regulation of β3-integrin, but further enhanced this effect (Fig. 3e, f). Thus, suppressing Spry4 increases β3-integrin protein independent of its regulation on RTK mediated MEK/MAPK or PI3 K/Akt signaling pathways in MDA-MB-231 cells.

### Suppression of Spry4 increases the CD133^+^ subpopulation and enhances tumorigenic potential of MDA-MB-231 cells

**Fig. 4:**
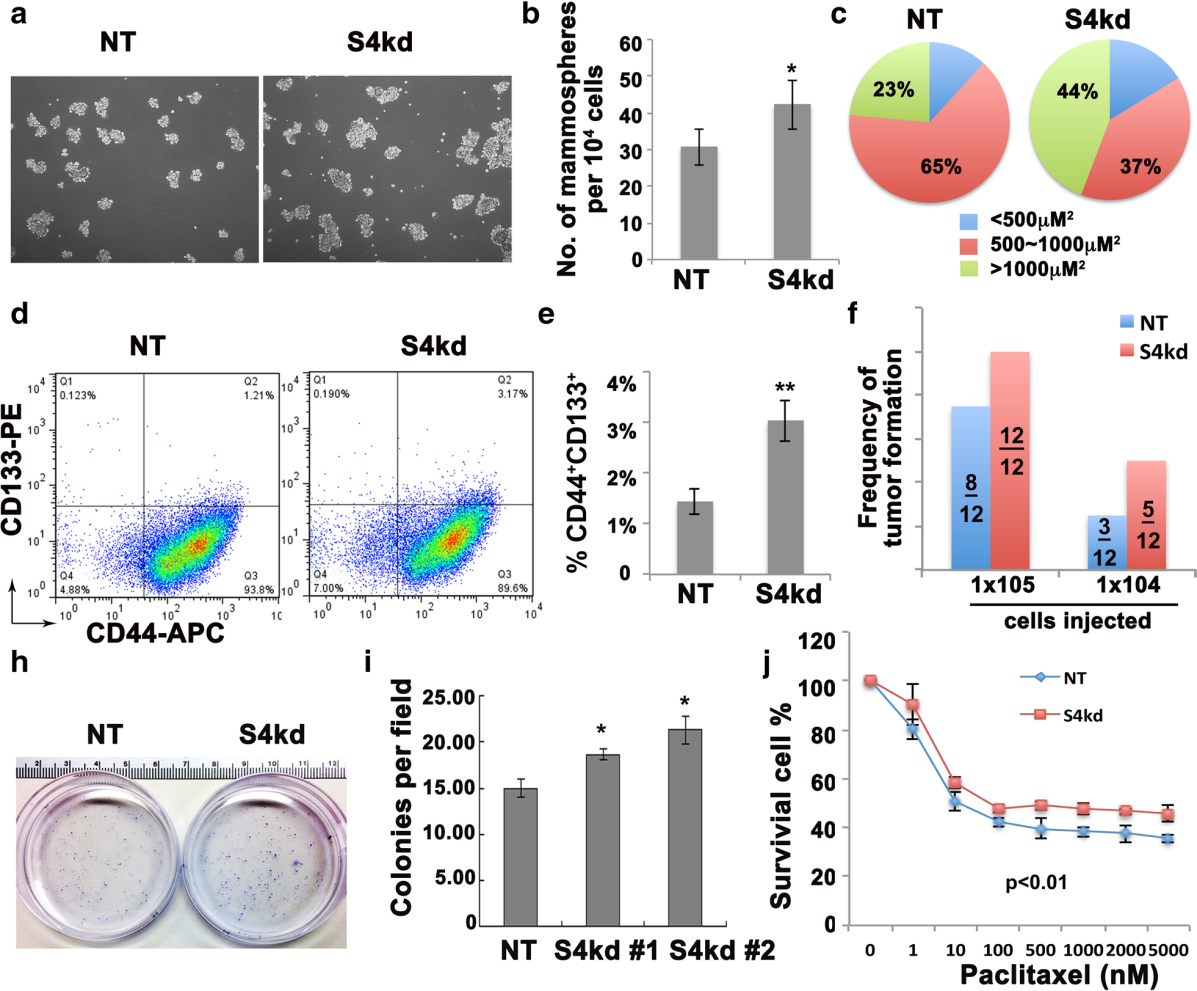
Suppressing Spry4 expression enhances cancer stem cell subpopulation and cancer stem cell features of MDA-MB-231 cells. **a** Representative images from mammosphere assays show S4kd cells formed more and larger mammospheres. **b** Quantification of mammosphere assays. **c** Allocation of different sized mammoshperes. **d** Representative results of FACS analysis of CD44 and CD133 expression. **e** Quantification of CD133^+^CD44^+^ population from three independent experiments. **f** In vivo LDA of tumor initiating capacity shows that S4kd MDA-MB-231 cells had higher chance to form palpable tumor compared to NT cells in vivo. **h** Representative images of clonogenic analysis of NT and S4kd MDA-MB-231 cell upon single high dosage Paclitaxel treatment. **i** Quantification of colonies formed from three independent experiments. **j** NT and S4kd cells were treated with Paclitaxel at an increasing dosage from 0 to 5 μM, survival cells were estimated with MTT measurement. The Paclitaxel killing curve shows that suppressing Spry4 decreased the sensitivity of MDA-MB-231 cell to Paclitaxel treatment. *p < 0.05

Integrin-β3 is a known mammary stem/progenitor cell marker, and also serves as a CSC marker [20]. Therefore, we hypothesized that MDA-MB-231 cells with suppressed Spry4 acquired more CSC features. Using a mammosphere forming assay, we observed that S4kd cells formed more and larger mammospheres compared with NT (Fig. 4a–c). CSCs in breast cancer have been characterized as CD44^+^/CD24^−^, and/or positive for aldehyde dehydrogenase 1 (ALDH1) [21, 22]. However, MDA-MB-231 cells are mesenchymal-like breast cancer cells, and majority of them express high levels of CD44 protein and are CD24 negative. Instead, we performed FACS to examine the expression of CD44 combined with CD133, another stem cell marker for normal or cancerous cell types [23, 24]. The results show that knockdown of Spry4 increased the number of CD133^+^CD44^+^ cells (Fig. 4d, e). One fundamental role of CSC is to initiate tumors in vivo. Therefore, we used a limiting dilution assay to examined whether suppressing Spry4 enhances the ability of MDA-MB-231 cells to form tumors in vivo. Eight-week old NOD/SCID females were injected with 1 × 10^3^, 1 × 10^4^ or 1 × 10^5^ non-targeting or S4kd MDA-MB-231 cells, and monitored for palpable tumor formation every 2 days. Within a 4-month experimental period, no mice injected with 1 × 10^3^ cells formed palpable tumors. However, at dosages of 1 × 10^4^ or 1 × 10^5^, mice injected with S4kd MDA-MB-231 cells had higher incidence of tumor formation than those injected with the same number of non-targeting cells (Fig. 4f). These data indicate that suppressing Spry4 increases tumor initiating potential of MDA-MB-231 cells. Collectively, our results indicate suppressing Spry4 expression may enrich the CSC subpopulation in MDA-MB-231 cells.

### Suppressing Spry4 expression decreases the sensitivity of MDA-MB-231 cells to Paclitaxel treatment

Drug resistance is a feature attributed to CSCs, and is a serious obstacle to cancer therapy [3, 4]. Since suppression of Spry4 enhances the CSC phenotype, we tested cell sensitivity to Paclitaxel, a common therapy for breast cancer treatment. In clonogenic assays, S4kd cells formed more and larger colonies following a single high dosage of Paclitaxel treatment compared to NT cells (Fig. 4h, i). Measurement of cell viability using the MTT assay also showed that suppressing Spry4 decreased the sensitivity of MDA-MB-231 to Paclitaxel treatment in a range from 0.001–5 μM and increased cell survival after 24 h of treatment. Paclitaxel had higher killing potential against NT than S4kd cells (Fig. 4j). These results suggest that endogenous Spry4 in human breast cancer MDA-MB-231 cells contributes to drug sensitivity.

## Discussion

CSCs play critical roles in cancer progression and metastasis. Spry4 has been shown to function as tumor suppressor [9–11]. The objective of this study was to test whether the suppressive role of Spry4 in tumorigenesis involves modulation of CSCs. Using the MDA-MB-231 model, we demonstrate that suppressing endogenous Spry4 increased cell growth and migration in vitro, xenograft tumor growth and metastasis in vivo, and these effects were accompanied by an increase in β3-integrin expression. We demonstrate that Spry4 knockdown MDA-MB-231 cells led to enhancement of CSC features, including increased CD133^+^CD44^+^ subpopulation and mammosphere formation, decreased sensitivity to Paclitaxel treatment in vitro, and increased capacity for xenograft tumor initiation in vivo. Thus, our results for the first time demonstrate a role of Spry4 in modulating CSC phenotype in the MDA-MB-231 breast cancer cell model.

RTK signaling not only regulates normal embryonic stem cells, but also plays important roles in acquisition and maintenance of CSCs in many cancers including glioblastoma, breast, head and neck squamous cell carcinomas [6, 8, 25–27]. The MAPK/ERK and PI3K/Akt signaling pathways play important roles in maintaining the “stemness” of normal and CSCs. Spry family proteins function as RTK signaling modulators and regulate stem cell self-renewal, survival and differentiation [28–31]. Our findings suggest that Spry4 also regulates CSCs, and this effect may not be restrained to MDA-MB-231 cells because MAPK/ERK and PI3 K/Akt pathways are shared in different cell types. In fact, we performed a similar Spry4 knockdown analysis in HTB-126, another breast cancer cell line, and found a similar increase of CSC properties in those cells (Additional file 1: Figure S2). Further study is warranted to evaluate whether this function of Spry4 is broadly conserved in multiple cancer types and stages of progression.

The mechanism of Spry4 in regulating tumor cell migration remains unclear. Expression of integrins is correlated with disease progression and metastasis in various tumor types including lung, melanoma and breast [10, 15, 32–36]. In MDA-MB-231 cell overexpression of β3-integrin promotes cell migration and invasion in vitro, and xenograft tumor cell lung metastasis in vivo [37]. Expression of β3-integrin has also been reported to promote spontaneous metastasis of breast tumors to bone [15, 38–40], and serves as a marker of CSCs in some murine [20] and human [41] breast tumors. The expression of integrins is also critical for mammary stem cell/progenitor behavior [42, 43] and breast carcinogenesis [44]. Studies have shown that sustained activation of the Raf-MEK-ERK signaling pathway induced expression of β3-integrin is associated with transformed cell [45]. PI3K/Akt signaling has also been shown to mediate IL-8 induced αvβ3 expression and motility in human chondrosarcoma cells [46]. MDA-MB-231 cells harbor an activating mutation in Ras, suppressing Spry4 expression had mild but significant increase on pERK activation, and chemical inhibition of MEK/MAPK signaling did not eliminate the increase of β3-integrin due to suppressing Spry4. We also observe an increase of pAkt with loss of Spry4 expression in MDA-MB-231 cells, however chemical inhibition of PI3K/pAkt signaling by PI3 K inhibitor did not normalize the expression of β3-integrin in S4kd cells. We have shown that Spry4 regulates β3-integrin degradation in endothelial cell by inhibiting VEGFR mediated Src activation [16], however, suppressing Spry4 in MDA-MB-231 cells appears to have no effect on Src activation when cells are cultured in growth medium. Additional study of how Spry4 regulates β3-integrin expression, and further examination whether acquisition of β3-integrin is necessary for the enhanced CSC phenotype of Spry4 knockdown cells is importance for better understanding CSC biology.

## Additional file

10.1186/s12935-016-0292-7 Suppression of Spry4 promotes cancer stem cell properties in breast cancer cell lines.
