# Erratum to: Suppression of Spry4 enhances cancer stem cell properties of human MDA-MB-231 breast carcinoma cells

**DOI:** 10.1186/s12935-017-0423-9

**Published:** 2017-05-11

**Authors:** Hongyu Jing, Lucy Liaw, Robert Friesel, Calvin Vary, Shucheng Hua, Xuehui Yang

**Affiliations:** 1 Center for Molecular Medicine, Maine Medical Center Research Institute, 81 Research Drive, Scarborough, ME 04074 USA; 2 Department of Respiratory Medicine, The First Hospital of Jilin University, Changchun, 130021 Jilin Province China

## Erratum to: Cancer Cell Int (2016) 16:19 DOI 10.1186/s12935-016-0292-7

Upon publication of the original article [1], it was noted that the Core Facilities utilized by the authors for their research were not adequately acknowledged in the manuscript. In this case, the following was mistakenly omitted:

- Progenitor cell analysis core
- Molecular Phenotyping core
- Stem Cell COBRE Grant P30GM106391
- Vascular Biology COBRE Grant P30GM103392

This has since been acknowledged and corrected in this erratum.

## References

[1] Jing H, Liaw L, Friesel R, Vary C, Hua S, Yang X (2016). Suppression of Spry4 enhances cancer stem cell properties of human MDA-MB-231 breast carcinoma cells. Cancer Cell Int.

