# Supplementary material for: Suppression of Spry4 enhances cancer stem cell properties of human MDA-MB-231 breast carcinoma cells
Source: Cancer Cell Int. 2016 Mar 11;16:19. doi: 10.1186/s12935-016-0292-7 (PMC4787021; doi:10.1186/s12935-016-0292-7)
Supplement: Supplementary file 1 — 10.1186/s12935-016-0292-7 Suppression of Spry4 promotes cancer stem cell properties in breast cancer cell lines. [file 12935_2016_292_MOESM1_ESM.docx]

Additional file 1:

**Suppression of Spry4 promotes cancer stem cell properties in breast cancer cell lines**

Additional file Figure S1.


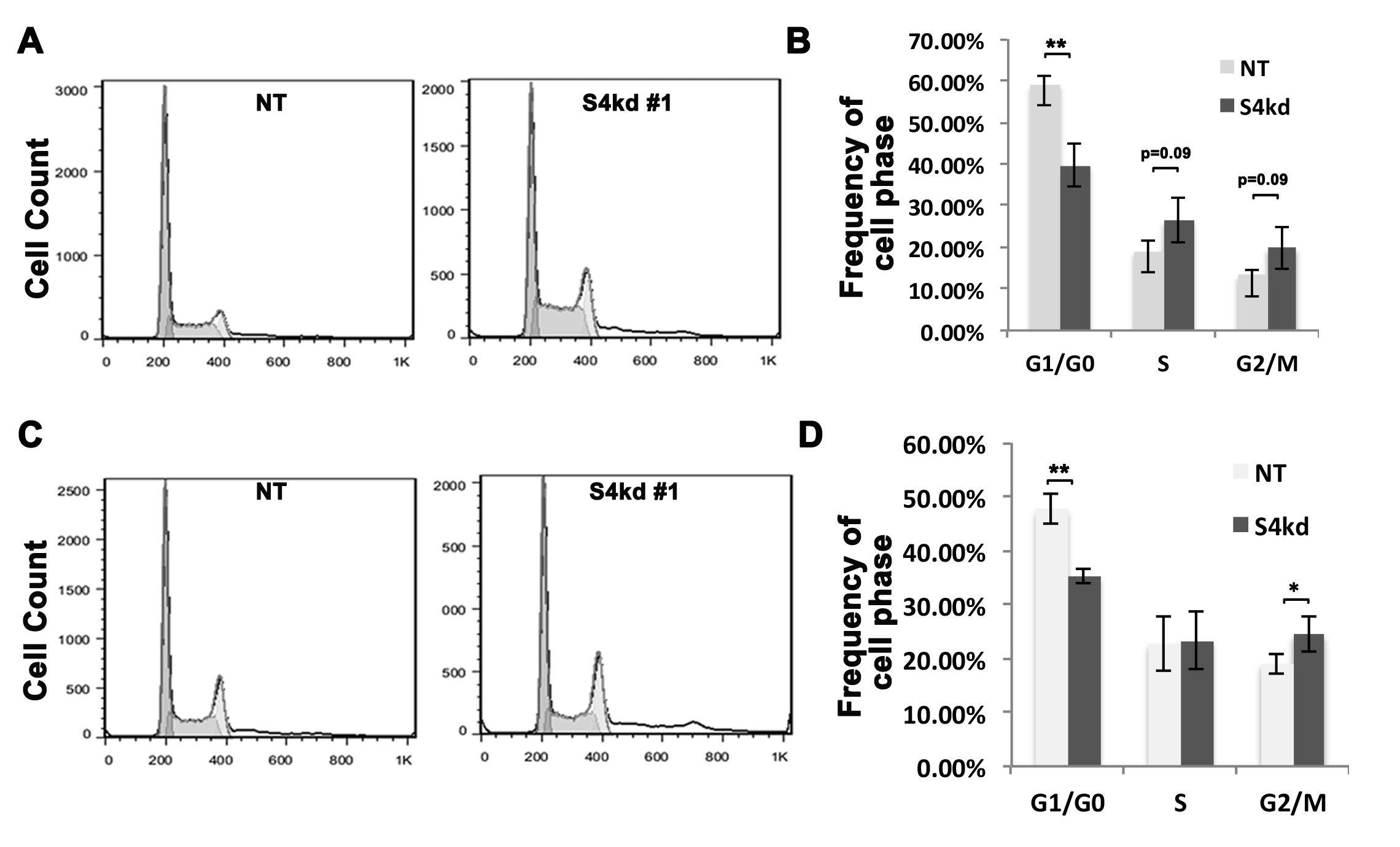


Additional file Fig. S1. **Suppressing Spry4 increases cell proliferation.** A) Representative cell cycle analysis of NT and S4kd#1 cells cultured in serum-free condition by FACS. B) Quantification of cell cycle analysis from three independent experiments shows that suppressing Spry4 decreased G1/G0 phase, and slightly increased S and G2/M cell populations when cells were cultured in serum-free condition. C) Representative cell cycle analysis of NT and S4kd#1 cells cultured in complete medium by FACS. D) Quantification of cell cycle analysis from three independent experiments shows that suppressing Spry4 decreased G1/G0 phase, and increased S and G2/M cell populations when cells were cultured in growth medium.

Additional file Figure S2


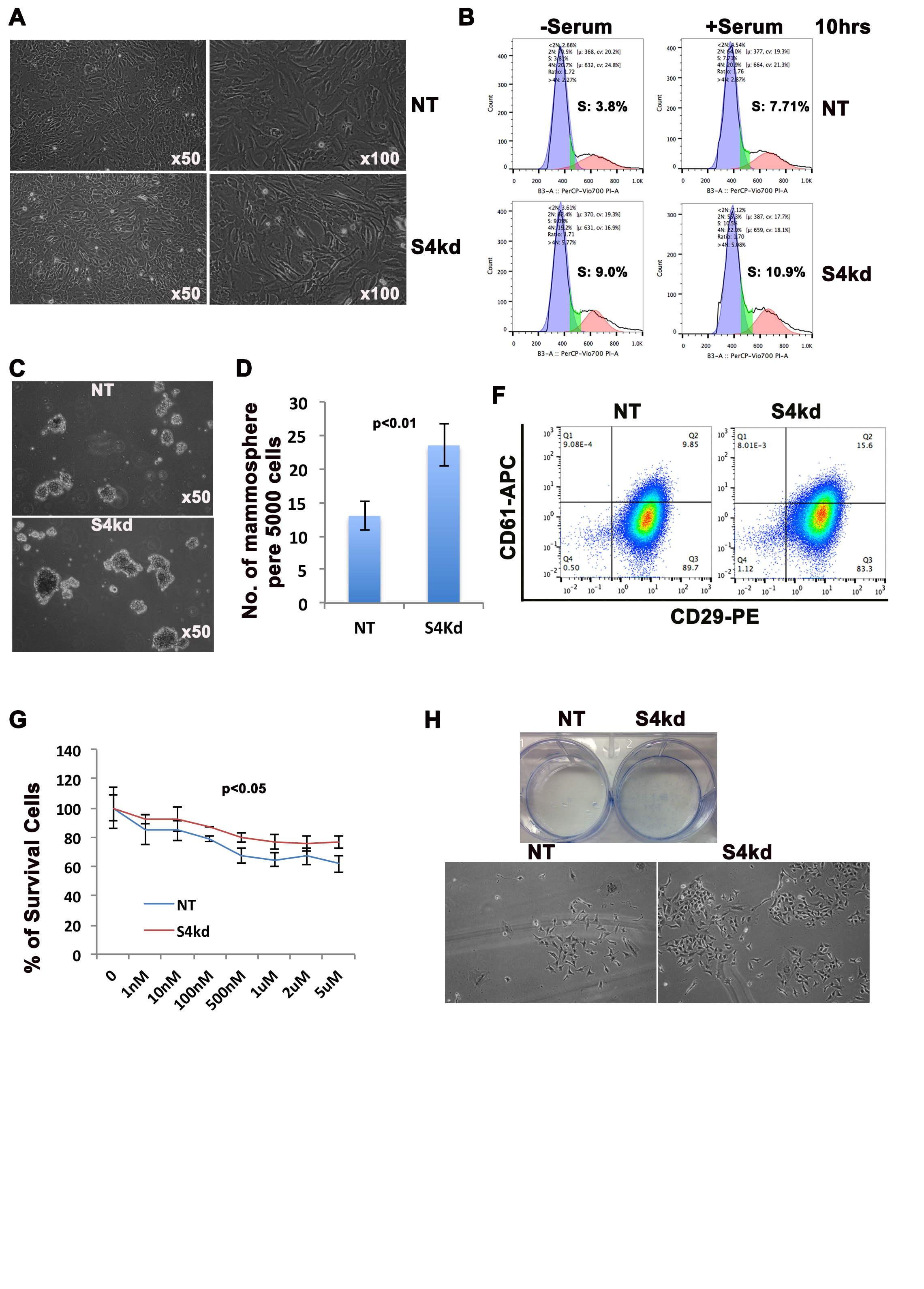


Additional file Fig. S2. **Suppression of endogenous Spry4 increases CSC properties in breast cancer cell line HTB-126.** HTB-126 cells were transduced with NT or hSpry4 shRNA lentiviruses, and selected in 2mg/ml puromycin for two weeks to generated NT or S4kd, respectively. A) Phage contrast image shows S4kd cells exhibit more fibroblastic-like morphology compared to NT cells. B) Cell cycle analysis. C) Representative images of mammosphere analysis. D) Quantification of formed mammospheres. E) FACS analysis of CD61 (integrin beta 3) and CD29 (integrin beta 1). F) Examination of the sensitivity of NT or S4kd cells in response to Paclitaxel treatment. G) Clonogenic analysis of NT or S4kd in response to a single high dosage (2μM) of Paclitaxel treatment.

Additional file Figure S3


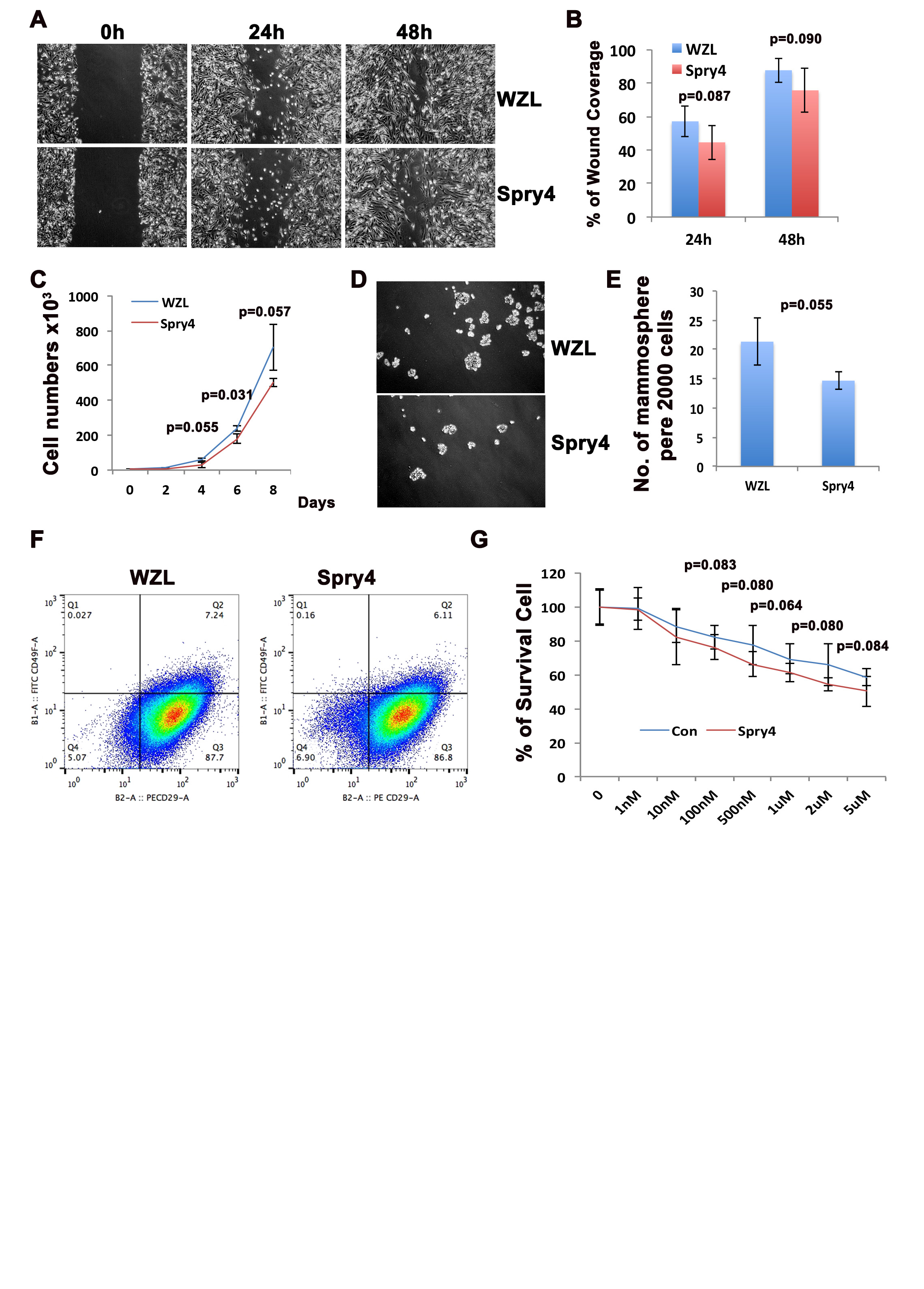


Additional file Fig. S3. **Overexpression of Spry4 has mild inhibition of MDA-MB-231 cells CSC properties.** MDA-MB-231 cells were transduced with mouse Spry4 (mSpry4) or WZL retroviruses, and selected in 100μg/ml hygromycin for two weeks to generate mSpry4 or WZL control stable cells. A) Wound healing analysis was used for examining the effect of overexpressing Spry4 on MDA-MB-231 cell migration. B) Quantification of wound coverage. C) Growth curve analysis of mSpry4 and WZL control MDA-MB-231 cells. D) Representative images of mammosphere formation assay after two weeks. E) Quantification of the number of formed mammospheres. F) FACS analysis of CD49f (integrin alpha 6) and CD29 (integrin beta 1) expression. G) Examination of the sensitivity of mSpry4 or WZL control cells in response to Paclitaxel treatment.
